# Preserving Airway Smooth Muscle Contraction in Precision-Cut Lung Slices

**DOI:** 10.1038/s41598-020-63225-y

**Published:** 2020-04-15

**Authors:** Guang Li, Jonathan A. Cohen, Carolina Martines, Sumati Ram-Mohan, Joseph D. Brain, Ramaswamy Krishnan, Xingbin Ai, Yan Bai

**Affiliations:** 1 Department of Critical Care Medicine, Renmin Hospital and Wuhan University, Wu Han, 430071 China; 2 Pulmonary and Critical Care Medicine, Brigham and Women’s Hospital and Harvard Medical School, Boston, MA 02115 USA; 3 Center for Vascular Biology Research, Department of Emergency Medicine, Beth Israel Deaconess Medical Center, Boston, MA 02215 USA; 4 Molecular and Integrative Physiological Sciences Program, Department of Environmental Health, Harvard T.H. Chan School of Public Health, Boston, MA 02115 USA; 5 Department of Pediatrics, Massachusetts General Hospital and Harvard Medical School, Boston, Boston, MA 02114 USA

**Keywords:** Biological techniques, Physiology

## Abstract

Precision-cut lung slices (PCLS) are ideal for measuring small airway contraction. However, these measurements are currently limited to acute exposure scenarios that typically last a few minutes to a few hours. Using an insulin-supplemented culture medium, we prolong the small airway contractility in mouse PCLS for up to two weeks. Compared to conventional culture medium, insulin-supplemented culture medium provides no additional benefit in preserving cellular viability or airway structure. However, it protects the airway smooth muscle (ASM) against a loss of smooth muscle myosin heavy chain (SMMHC) expression. We elucidate the significance of this new culture medium for chronic disease modeling of IL-13-induced airway hyper-responsiveness.

## Introduction

A powerful tool for studying airway reactivity is the preparation of precision-cut lung slices (PCLS). In PCLS, small airway contraction can be readily imaged^1^, while corresponding changes in mechanotransduction and airway smooth muscle contraction are precisely quantified^2–4^. Neural stimulation and dynamic stretching can also be superimposed^5–8^. PCLS can be prepared from naïve, genetically modified, or diseased animals as well as from human surgical samples and lung explants, and even cryopreserved for future use^9,10^. Thus, they are ideally suited to bridge the translational gap between cell culture models and whole animal or human subject studies. Given its unique advantages, PCLS have rapidly emerged as an important platform for airway reactivity studies that typically last a few minutes to a few hours^11–15^.

PCLS are typically cultured in DMEM/F12 medium (1:1, DMF12). This medium preserves cell viability and airway structure in the mouse PCLS for 3–7 days^1^ and in the human PCLS for approximately 2 weeks^16^, during which PCLS have been applied widely to study chemical toxicity^16–18^, chemical or cytokine-induced fibrosis^19,20^, innate immunity to viral and bacterial infection, or pro-inflammatory stimuli^18,21^ and acute airway responses. However, the full potential of PCLS culture in lung research has been restricted by its relatively short survival *in vitro*. For instance, in the PCLS model of idiopathic pulmonary fibrosis (IPF)^19^, even though an early fibrosis-like change was induced within 7 days, a longer duration was required to establish robust and irreversible extracelluar matrix changes. Similarly, in the PCLS model of alveologenesis^22^, a prolonged culture was necessary to examine septation at later developmental stages. Likewise, to evaluate inflammatory and innate immune responses to infection^18^, two-week-long PCLS culture was needed to fully evaluate the impact of infection on cellular metabolism and structural integrity. Lastly, airway contractility has rarely been quantified beyond 72 hours in culture. This poses a critical impediment to the investigation of airway contractile/relaxant mechanisms in longitudinal studies, especially in the context of chronic disease modeling. A recent technical breakthrough has improved the viability of PCLS culture by embedding the slices in engineered hydrogel biomaterials^23^. However, whether the biomaterial maintains airway contractility in PCLS has yet to be tested. Moreover, the hydrogel plug inside the lumen may bring resistance against contraction making the model less suitable for the airway function study.

To preserve airway contraction in PCLS, we test insulin supplementation to the conventional DMEM/F12 (DMF12) medium. We show that this newly formulated insulin supplemented DMF12 (DMF12-Ins) medium preserves contractile proteins in airway smooth muscle cells and prolongs the airway contractile function in the mouse PCLS for at least two weeks. This improved PCLS culture facilitates chronic disease modeling, such as Interleukin (IL)-13-induced airway hyper-reactivity.

## Results

### Insulin prolongs airway contraction in PCLS for two weeks

**Figure 1:**
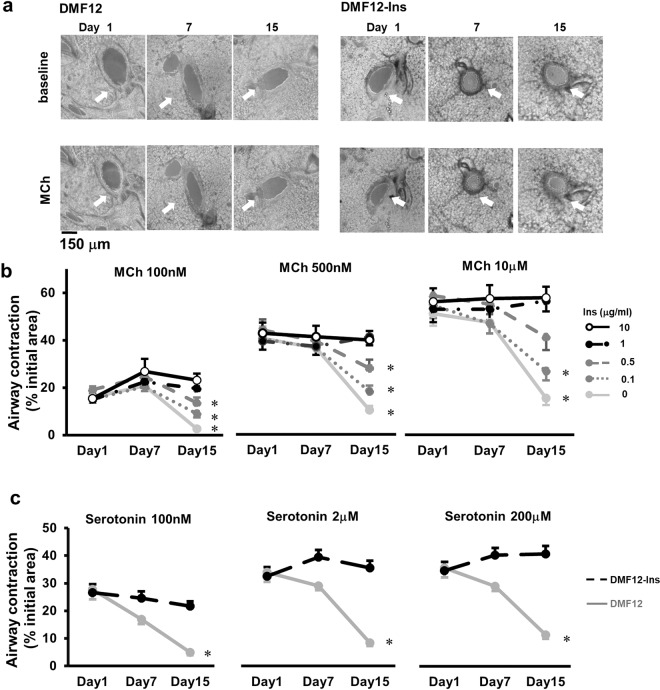
Insulin prolongs the airway responsiveness of PCLS. (**a**) Representative images of airways (arrows) in response to 500 nM MCh on days 1, 7, and 15 of the PCLS culture. The conventional DMF12 is shown in the left panels and the modified DMF12-Ins (with 1 μg/ml insulin) group is shown in the right panels. The dose-response of airways to MCh (**b**) and serotonin (**c**) in the conventional DMF12 culture or modified culture of DMF12 with supplemental 0.1–10 μg/ml insulin. Each point represents the mean ± SEM of 8–25 slices from 4 mice. Data were analyzed with two-way ANOVA followed by Sidak’s or Tukey’s test. *p < 0.05 as compared to the 1 μg/ml insulin group on day 15. At all 3 concentrations of MCh and serotonin, a significant interaction between insulin and the culture time impacted the airway contractile responses. At the tested insulin concentrations, we found 1 and 10 μg/ml insulin maintained airway contraction throughout the entire culture period.

Insulin is a critical growth factor for airway smooth muscle (ASM) by promoting the contractile phenotype in primary cell culture^24^. However, its impact on ASM contractility in PCLS has not been adequately characterized. Here, we carefully examined insulin supplementation to the PCLS culture. We found that in response to MCh and serotonin, PCLS in the absence of insulin gradually lost airway reactivity after day 7. Strikingly, the PCLS culture in the presence of 1 μg/ml insulin exhibited robust airway responsiveness that was unchanged for 15 days (Fig. 1a–c). These results show that insulin has a beneficial effect on the maintenance of airway contractility of PCLS.

To evaluate whether the effect of insulin on airway contractility of PCLS was dose-dependent, we examined the airway responsiveness of the PCLS culture supplemented with insulin at concentrations of 0.1–10 µg/ml (Fig. 1b). First, we confirmed that insulin did not trigger any acute airway response at any tested concentration (data not shown). Next, we assessed airway contraction to MCh in insulin-supplemented PCLS culture at days 1, 7, and 15. We found that airway contractility was preserved by insulin in a dose-dependent manner (Fig. 1b). Insulin at concentrations of 0.1 µg/ml and 0.5 µg/ml partially prevented the gradual loss of airway contractility in culture, as a significant reduction in MCh-induced airway contraction was observed over time in both culture groups (0.1 µg/ml insulin group: F = 20.94, p < 0.001; 0.5 µg/ml insulin group: F = 7.15, p = 0.002) (Fig. 1b). However, at the concentration of 1 µg/ml, insulin fully maintained airway contraction in response to MCh for as long as 15 days (F = 0.30, p = 0.74) (Fig. 1b). Insulin at higher concentrations, such as 10 µg/ml, had a similar beneficial effect (F = 0.20, p = 0.81) as insulin at 1 µg/ml. We thus chose insulin at 1 µg/ml in the DMF12-Ins culture for subsequent studies.

### DMF12-Ins medium does not confer an additional benefit to PCLS viability or metabolism

**Figure 2:**
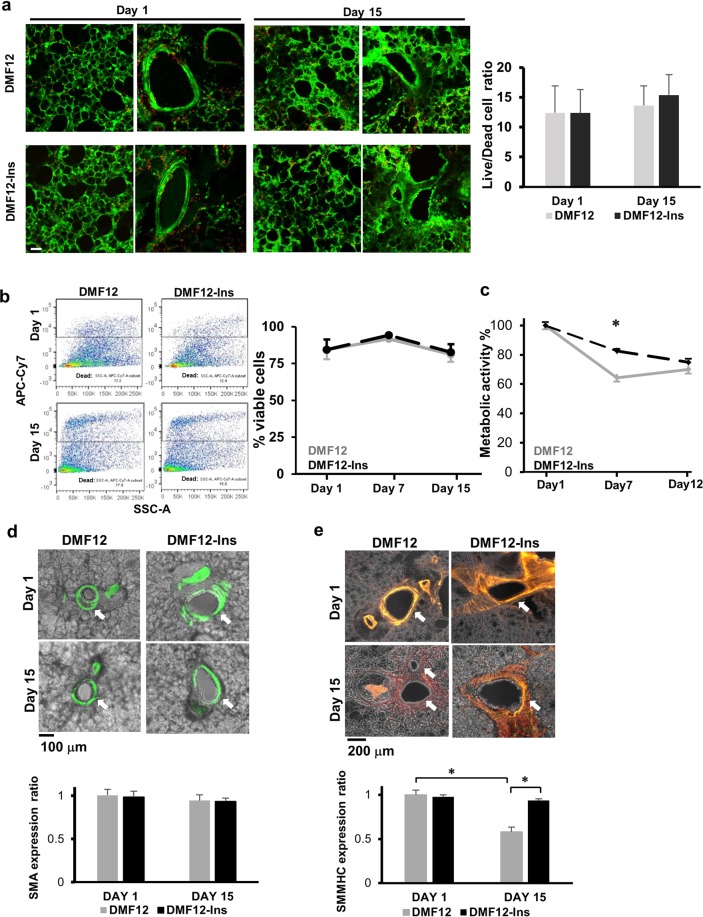
The DMF12-Ins medium has no effect on the viability of PCLS but preserves smooth muscle myosin heavy chain (SMMHC) expression in ASM. (**a**) Representative confocal images of live-dead stained PCLS (left panel) and a summary plot of living to dead cell ratio (right panel) in PCLS from the conventional DMF12 group and the new DMF12-Ins group at days 1 and 15. Dead cells are labeled with red fluorescence. Scale bar = 50 μm. Each column represents the mean ± SEM of 6–7 slices from 3 mice. (**b**) Representative flow cytometry analyses and summary plot of viable cell percentage at days 1 and 15 in DMF12 and DMF12-Ins groups. Each measurement represents the mean ± SEM of 4 independent experiments. (**c**) Colorimetric cell metabolic analyses of PCLS at days 1, 7, and 12 in two culture conditions. Each dot represents mean ± SEM of 14 slices from 4 mice. *p < 0.05 by Sidak’s test following two-way ANOVA. (**d**) Representative images (upper panel) of the PCLS prepared from *SMA-GFP* transgenic mice and the summary plot of airway SMA fluorescent intensity (lower panel) on days 1 and 15 in the DMF12 and DMF12-Ins groups. (**e**) Representative SMMHC immunofluorescent images (upper panel) and the summary plot of fluorescence intensity (lower panel) on days 1 and 15 in the DMF12 and DMF12-Ins groups. The expression level of SMA and SMMHC was presented as a ratio to the average level of the respective protein in the DMF12 PCLS culture on day 1. The arrow marks the airway. The bar graph represents mean ± SEM of 7–8 slices from 4 mice. *p < 0.05, by Tukey’s test following 2-way ANOVA.

We tested whether insulin supplementation preserved airway responsiveness by improving cell survival, especially of ASM cells in PCLS. We compared cell viability in PCLS culture in either DMF12 or DMF12-Ins medium, using two different methods. Using a live/dead staining method, we found that only a small percentage of cells in PCLS were dead even at day 15 in both conventional and DMF12-Ins cultures (Fig. 2a). In addition, a majority of dead cells in PCLS were alveolar and airway epithelial cells, but not ASM cells (Fig. 2a). Similarly, using flow cytometry to quantify live cells in the single-cell suspension dissociated from PLCS, we found low percentages (12–17%) of eFluor 780+ dead cells at days 1, 7, and 15 in both DMF12 and DMF12-Ins culture conditions (Fig. 2b). Because enzymatic dissociation of PCLS itself causes cell death, the actual extent of cell death in the PCLS culture was likely lower than the measurement by flow cytometry. Microscopically, the structure of the airway was intact for at least 15 days in both PCLS cultures (Fig. 2a). These findings indicate that the preservation of airway responsiveness in PCLS conferred by the DMF12-Ins culture medium is not due to an improvement of cell viability, although maintaining cell viability is critical for ASM contraction.

Next, we tested whether insulin supplementation preserved airway responsiveness by improving cell metabolism in PCLS. Using a colorimetric MTS assay, we found the metabolic enzyme activity of PCLS in DMF12 culture decreased by ~40% within the first week before stabilizing at ~65% of the initial level after day 7 (Fig. 2c). In comparison, the DMF12-Ins culture exhibited a slower decline in the metabolic enzyme activity but ultimately reached a similar level as the conventional culture at day 12 (Fig. 2c). Taken together, insulin supplementation has no effect on the overall cell viability, histological appearance, or metabolic enzyme activities of PCLS.

### DMF12-Ins uniquely maintains the expression of a key ASM contractile protein in PCLS

Preserving the expression of contractile proteins, such as smooth muscle myosin heavy chain (SMMHC), is crucial in maintaining ASM contractility and subsequent airway constriction. We thus hypothesized that the DMF12-Ins medium prolonged airway responsiveness in PCLS by preserving the expression of key ASM contractile proteins. To test this hypothesis, we compared PCLS in DMF12 vs. DMF12-Ins culture conditions for α-smooth muscle actin (SMA) and SMMHC expression. For assessment of α-SMA expression, we took advantage of an *SMA-GFP* reporter line. We found comparable green fluorescence in the ASM of PCLS in both DMF12 and DMF12-Ins culture conditions on days 1 and 15 (Fig. 2d); thus, insulin supplementation does not impact the expression of α-SMA. This finding is also consistent with our previous finding that there is no significant ASM cell death in PCLS over time (Fig. 2a–c). In contrast, SMMHC expression in ASM cells was maintained only in the DMF12-Ins culture for 15 days (0.98 ± 0.07 on day 1 as compared to 0.94 ± 0.03 on day 15) and the level of SMMHC reduced in the DMF12 culture by almost 50% (0.98 ± 0.07 on day 1 as compared to 0.57 ± 0.03 on day 15) (Fig. 2e). These findings indicate that the new DMF12-Ins culture preserves airway contraction possibly by maintaining the expression of key contractile proteins in ASM in PCLS.

### DMF12-Ins sustains IL-13-induced airway hypercontractility in PCLS culture

**Figure 3:**
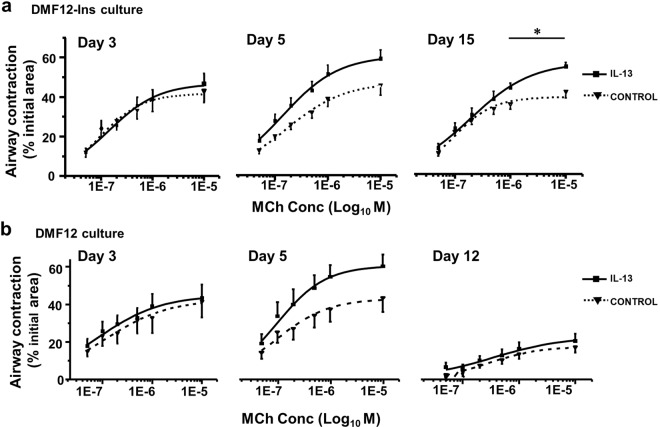
IL-13 treatment induces sustained airway hypercontractility in DMF12-Ins PCLS culture. The effect of IL-13 on MCh-induced airway contraction was assayed (**a**) in the DMF12-Ins PCLS culture on days 3, 5, and 15 (n = 11–16 slices from 4 mice), and (**b**) in the DMF12 PCLS culture on culture days 3, 5, and 12 (n = 6–11 slices from 4 mice). IL-13 treatment increased airway contractility on day 5 in the DMF12-Ins (p < 0.05) and the DMF12 group (p = 0.05) by two-way ANOVA. The IL-13 induced airway hypercontractility persisted in the DMF12-Ins group on day 15. p < 0.05 by two-way ANOVA, *p < 0.05 by the subsequent Sidak’s test showing the airway reaction significantly different between control and IL-13 treatment at the indicated (*) MCh concentration group. In comparison, the MCh-response reduced in the DMF12 PCLS culture regardless of IL-13 treatment.

Taking advantage of prolonged airway responsiveness in the PCLS DMF12-Ins culture, we tested whether PCLS may be utilized to model chronic airway hyperresponsiveness, a hallmark of allergic asthma. To do so, we treated the PCLS cultured in DMF12-Ins medium with 25 ng/ml IL-13. IL-13 treatment induced airway hyperresponsiveness to MCh as early as day 5 (F = 5.097, p = 0.03). IL-13-induced airway hypercontractility was maintained in the PCLS DMF12-Ins culture up to day 15 (F = 5.33, p = 0.03) (Fig. 3a). In comparison, even though the PCLS culture in the DMF12 medium developed similar IL-13-induced airway hypercontractility on day 5 (F = 4.33, p = 0.05), it gradually lost airway contractility irrespective of the presence of IL-13 (F = 0.77, p = 0.39) (Fig. 3b). These findings indicate that while IL-13 is able to acutely induce airway hyperresponsiveness, it fails to maintain the contractile phenotype of ASM over an extended period of time. Taken together, the PCLS culture in the new DMF12-Ins medium provides an appropriate time window for mechanistic investigation and pharmaceutical exploration of an established hypercontractile phenotype of ASM.

## Discussion

In this study, we have developed a new culture medium that preserves airway contractility in PCLS. We illustrate its utility in a prolonged model of IL-13-induced airway hyperresponsiveness.

Mouse PCLS have been primarily applied to investigate the biological responses to acute stress. To expand its frontiers to chronic assays, research groups have attempted to prolong the survival of PCLS by optimizing nutritional support with a variety of culture media, such as MEM^25,26^, DMEM^27–29^, DEME/F12^2,4,30^, RPMI-1640^9,31^, and M199^32,33^. Nevertheless, thus far, no specific medium has been found to provide any survival benefit. In this regard, neither did our newly proposed DMF12-Ins medium. Indeed, we found that the functional loss of ASM contraction preceded cell death in the mouse PCLS. Insulin supplementation is known to preserve/promote the contractile phenotype of cultured human ASM cells^24,25^. Hence, using an insulin supplement to preserve the expression of contractile proteins in ASM provides a logical solution to maintain airway function in PCLS. We demonstrate here that insulin supplementation preserves the expression of airway smooth muscle myosin heavy chain (SMMHC) in the mouse PCLS. Mechanistically, insulin may activate the Rho/Rho kinase and PI-3 kinase/Akt1/mTOR pathways to maintain the SMMHC synthesis in ASM cells^25,26^. Insulin may similarly induce the expression of laminin, an extracellular matrix protein that is involved in the maintenance of contractile ASM phenotype^27^.

Insulin was reported to regulate ASM contraction via inhibition of the neuronal M2 muscarinic receptor and acetylcholine release^34^. It was also found to downregulate the M2 muscarinic receptor on ASM, which counteracts the airway contraction upon activation^35^. However, in the present study, these mechanisms are unlikely applicable since the preservation of airway contractile responses is not limited to the muscarinic receptor agonist. Of note, despite its capacity to maintain airway contractility, insulin was not sufficient to induced airway hypercontractility at the tested concentrations. However, insulin has a dose-dependent effect on ASM^26,36,37^. Therefore, it is critical to choose an optimal insulin concentration to avoid the undesired impact of insulin on the ASM in PCLS cultures. In the present study, we confirmed that 0.1 to 10 µg/ml insulin had no significant side effects on the ASM size or triggered airway hyperreactivity in the mouse PCLS. Moreover, insulin at a concentration as low as 1 µg/ml preserved airway contractile function for up to 15 days, and it did not interfere with the regulatory role of IL-13 on airway contraction. Importantly, since DMF12-Ins medium requires no additional serum supplementation, this new culture of PCLS obviates any confounding effects of serum-borne hormones or growth factors that could lead to the phenotypic changes of ASM^38,39^.

The insulin effect on *in vivo* lung function has been examined in clinical trials on human patients^40–42^ and experiments on different animal species^43,44^, which yield no consistent findings. Inhaled insulin demonstrated reversible or non-progressive mild impact on the patient’s airflow rate, as indicated by the reduction of FEV1.0^40–42^, but had no significant influence on pulmonary function or lung histopathology in experimental dogs^43,44^. The discrepancy may be caused by the difference in dosage and duration of insulin treatment and species-specific airway anatomy that affects airway flow measurement.

A major advantage of preserving airway function in the DMF12-Ins culture of PCLS lies in the application of PCLS to model chronic airway disease *ex vivo*. In the present study, this application was demonstrated by using IL-13 treatment to simulate chronic airway contractile regulation in asthma. IL-13, at 20–100 ng/ml, was reported to induce acute ASM hypercontractility within 24 hours^45–47^. However, its long-term impact on the ASM, a more relevant clinical scenario in asthmatic patients, has never been directly assessed due to the lack of an appropriate model. In the present study, for long-term IL-13 exposure, we chose the concentration of 25 ng/ml, which may be more physiologically relevant than 100 ng/ml in the chronic phase of allergic airway inflammation. This might explain why it took longer to induce airway hypercontractility in our experimental setting. We showed that the IL-13 effect persisted for at least 15 days. This provides an *ex vivo* model to explore subsequent airway remodeling and to test potential pharmaceutic interventions to reverse the established airway phenotype. The DMF12-Ins PCLS culture serves as a supplementary approach in the study of chronic airway diseases and offers an additional advantage of reducing the number of experimental animals.

We acknowledge three limitations of our current work. First, insulin is a pleiotropic growth factor and may impact many cell types in PCLS^48^. This possibility warrants future investigation. Second, we have limited our investigation to the mouse PCLS. It remains to be seen if the DMF12-Ins medium would have ubiquitous benefits in PCLS from other species, especially human. Finally, the IL-13-treated PCLS model is a simplistic simulation of the chronic asthmatic lung pathophysiology and our work was focused on the reaction of ASM. IL-13 also impacts other cell types *in vivo*, especially epithelial cells^49^. Therefore, it would be of significant interest to consider/quantify airway epithelial changes and structural remodeling in PCLS as a supplementary assay to the animal model of chronic asthma.

In conclusion, by supplementing 1 µg/ml insulin to DMEM/F-12 medium, we have introduced a new culture method to preserve the contractile function of ASM in the mouse PCLS. This method uniquely enables longitudinal studies of IL-13 induced airway hyper-responsiveness *ex vivo*. More generally, it expands the range of applications for PCLS in mechanistic and interventional studies of airway contractile regulation.

## Materials and Methods

ARRIVE guidelines were followed when conducting and reporting the study.

### PCLS preparation

Male and female adult C57BL/6 mice were purchased from the Jackson Laboratory (Bar Harbor, ME). *αSMA-GFP* mice were provided by Dr. Alan Fine at the Boston University School of Medicine^50^. Protocols for mouse handling and euthanization were approved by the Institutional Animal Care and Use Committee at Brigham & Women’s Hospital, Harvard Medical School. Mouse lung lobes were inflated with 1.5% agarose-Hank’s buffered saline solution (HBSS) to total lung capacity (~1.2 ml) and sectioned using a vibratome (VF-310-0Z, Precisionary Instruments LLC, Natick, MA) to produce 150 µm thick PCLS. Additional details are provided elsewhere^2,4^.

### PCLS culture

PCLS were collected from the left mouse lung lobe, sorted serially during the slicing process. The adjacent lung slices were distributed sequentially to different cultural groups to reduce intergroup heterogeneity^17^. PCLS were cultured in DMF12 (1:1, ThermoFisher Scientific, Waltham, MA) or in DMF12 supplemented with 0.1, 0.5, 1, or 10 µg/ml insulin (Sigma-Aldrich, St. Louis, MO). Antibiotic-antimycotic (1×, ThermoFisher Scientific) was added to all culture media to prevent bacterial and fungal contamination during the entire culture period. For all experiments, the medium was changed every 48 hours. For IL-13-induced airway hyperreactivity, IL-13 (25 ng/ml, PerproTech, Roky Hill, NJ) was added to the PCLS culture in the DMF12 or DMF12-Ins medium from day 1.

### Airway contraction assay

The airways on PCLS were stimulated with either methacholine (MCh) or serotonin. Images of one constricting airway per PLCS were acquired at a 10×10 or 20×10 magnification using an inverted phase-contrast fluorescence microscope (Nikon Eclipse TS 100; Nikon, Tokyo, Japan) equipped with a Nikon DS-Ri2 camera. The airway lumen area was determined by tracing a contour around the airway using the magic wand tool of the NIH Image J software (National Institutes of Health, Bethesda, MD). Airway lumen area following treatment was normalized to the pre-treatment value to determine the extent of airway contraction. All airways in the current study had a pre-treatment diameter of 100–300 µm.

### Cell viability assay

Cell viability in PCLS was analyzed using two methods. One method was to label the PCLS using a LIVE/DEAD kit (L-3224; Molecular Probes, Eugene, OR), then acquire fluorescent images using a confocal microscope (Leica TCS SPE; Leica Microsystems, Wetzlar, Germany), and, finally, compute areas of live/dead cells using the NIH Image J software (National Institutes of Health, Bethesda, MD). The paraformaldehyde-fixed PCLS (all dead cells) were used as a control for this assay. The other method was to utilize flow cytometry analysis. For this method, 15–20 mouse slices were pooled and digested in 6 ml HBSS containing 400 U/ml Collagenase Type IV (Worthington Biochemical, Lakewood, NJ), 0.1% Dispase II (Roche, Indianapolis, IN), and 30 µL DNase I (New England Biolabs, Ipswich, MA). After 15 min at 37 °C, dissociated cells were filtered through a 100 µm cell strainer, stained with eFluor 780 (ThermoFisher Scientific) and analyzed using a flow cytometer (BD FACSCanto II, BD Biosciences, San Jose, CA). An unstained sample was used to set up the gate to distinguish live and dead cells.

### Metabolic enzyme activity assay

Metabolic enzyme activities were measured using CellTiter 96 AQueous One Solution Reagent (MTS, Promega, Madison, WI). Each slice was placed in 200 µl MTS reagent for one hour at 37 °C. The supernatant was collected, and the absorbance at 490 nm was measured using a SpectraMax M5 microplate reader (Molecular Devices, Sunnyvale, CA).

### Immunofluorescent staining

PCLS were fixed with 4% paraformaldehyde, stained with rabbit anti-smooth muscle myosin heavy chain 11 (anti-SMMHC, 1: 100, Abcam, Cambridge, MA) overnight at 4 °C, and then with Alexa Fluor 546 conjugated anti-rabbit (1:100, Thermo Fisher Scientific) for 1 hour at room temperature. The fluorescence-stained PCLS were imaged with the Nikon phase-contrast fluorescence microscope (Nikon Eclipse TS 100; Nikon, Tokyo, Japan). The mean fluorescent intensity of positively stained cells was measured with NIH Image J.

### Statistics

For airway contraction assay, each slice was considered as a separate sample and only one airway in each slice was recorded for the contraction assay. For the quantification of immunofluorescent intensity, 1–3 airways in one slice were measured and signals were averaged to represent one sample. To estimate the sample size that is sufficient for statistical analysis, we used an alternative resource equation approach^51^. No duplicate measurement was made. Data are represented as mean ± SEM. GraphPad Prism 8 was used for data analysis. Normal Gaussian distribution of the data was verified by the Kolmogorov-Smirnov test. For comparison between 2 groups, the statistical analyses were performed using the two-tailed Student’s t-test. For comparison of multiple groups involving 2 factors, the statistical analyses were performed with two-way ANOVA followed by Sidak’s or Tukey’s post hoc test as appropriate for multiple comparisons. Specially, we compared (1) the impact of insulin concentration and culture time on the MCh- (Fig. 1b) or serotonin-induced airway contraction (Fig. 1c); (2) the impact of culture time on the MCh or serotonin concentration-dependent contraction in each insulin concentration group; (3) the impact of insulin and culture time on the cell viability and metabolism (Fig. 2a-c); (4) the impact of insulin and culture time on the expression of SMA and SMMHC (Fig. 2d,e); (5) the impact of IL-13 treatment on the MCh concentration-dependent contraction in DMF12-Ins (Fig. 3a) and DMF12 culture group (Fig. 3b); and (6) the impact of culture time on IL-13-induced contractile regulation in DMF12 or DMF12-Ins groups (Fig. 3a,b). p < 0.05 was considered statistically significant.

## References

[1] Sanderson MJ (2011). Exploring lung physiology in health and disease with lung slices. Pulm Pharmacol Ther.

[2] Perez JF, Sanderson MJ (2005). The frequency of calcium oscillations induced by 5-HT, ACH, and KCl determine the contraction of smooth muscle cells of intrapulmonary bronchioles. J Gen Physiol.

[3] Ram-Mohan, S. *et al*. Tissue traction microscopy to quantify muscle contraction within precision-cut lung slices. *Am J Physiol Lung Cell Mol Physiol*, 10.1152/ajplung.00297.2019 (2019). 10.1152/ajplung.00297.2019 PMC7052683 31774304

[4] Bai Y, Sanderson MJ (2006). Modulation of the Ca2+ sensitivity of airway smooth muscle cells in murine lung slices. Am J Physiol Lung Cell Mol Physiol.

[5] Davidovich N, Chhour P, Margulies SS (2013). Uses of Remnant Human Lung Tissue for Mechanical Stretch. Studies. Cellular and molecular bioengineering.

[6] Lavoie TL (2012). Dilatation of the constricted human airway by tidal expansion of lung parenchyma. American journal of respiratory and critical care medicine.

[7] Patel KR, Bai Y, Trieu KG, Barrios J, Ai X (2017). Targeting acetylcholine receptor M3 prevents the progression of airway hyperreactivity in a mouse model of childhood asthma. FASEB J.

[8] Schleputz M, Uhlig S, Martin C (2011). Electric field stimulation of precision-cut lung slices. J Appl Physiol (1985).

[9] Bai Y (2016). Cryopreserved Human Precision-Cut Lung Slices as a Bioassay for Live Tissue Banking. A Viability Study of Bronchodilation with Bitter-Taste Receptor Agonists. Am J Respir Cell Mol Biol.

[10] Rosner SR (2014). Airway contractility in the precision-cut lung slice after cryopreservation. Am J Respir Cell Mol Biol.

[11] Bourke JE (2014). Novel small airway bronchodilator responses to rosiglitazone in mouse lung slices. Am J Respir Cell Mol Biol.

[12] Deshpande DA (2010). Bitter taste receptors on airway smooth muscle bronchodilate by localized calcium signaling and reverse obstruction. Nat Med.

[13] Mikami M, Perez-Zoghbi JF, Zhang Y, Emala CW (2019). Attenuation of murine and human airway contraction by a peptide fragment of the cytoskeleton regulatory protein gelsolin. Am J Physiol Lung Cell Mol Physiol.

[14] Koziol-White, C. J. *et al*. Soluble Guanylate Cyclase Agonists Induce Bronchodilation in Human Small Airways. *Am J Respir Cell Mol Biol*, 10.1165/rcmb.2019-0001OC (2019). 10.1165/rcmb.2019-0001OC PMC6938135 31340135

[15] Martin C, Uhlig S, Ullrich V (1996). Videomicroscopy of methacholine-induced contraction of individual airways in precision-cut lung slices. Eur Respir J.

[16] Neuhaus V (2017). Assessment of long-term cultivated human precision-cut lung slices as an *ex vivo* system for evaluation of chronic cytotoxicity and functionality. Journal of occupational medicine and toxicology (London, England).

[17] Watson CY (2016). Screening for Chemical Toxicity Using Cryopreserved Precision Cut Lung Slices. Toxicological sciences: an official journal of the Society of Toxicology.

[18] Temann A (2017). Evaluation of inflammatory and immune responses in long-term cultured human precision-cut lung slices. Human vaccines & immunotherapeutics.

[19] Alsafadi HN (2017). An *ex vivo* model to induce early fibrosis-like changes in human precision-cut lung slices. Am J Physiol Lung Cell Mol Physiol.

[20] Cedilak M (2019). Precision-cut lung slices from bleomycin treated animals as a model for testing potential therapies for idiopathic pulmonary fibrosis. Pulm Pharmacol Ther.

[21] Henjakovic M (2008). *Ex vivo* testing of immune responses in precision-cut lung slices. Toxicol Appl Pharmacol.

[22] Pieretti AC, Ahmed AM, Roberts JD, Kelleher CM (2014). A novel *in vitro* model to study alveologenesis. Am J Respir Cell Mol Biol.

[23] Bailey KE (2020). Embedding of Precision-Cut Lung Slices in Engineered Hydrogel Biomaterials Supports Extended *Ex Vivo* Culture. Am J Respir Cell Mol Biol.

[24] Hayashi K (1998). Differentiated phenotype of smooth muscle cells depends on signaling pathways through insulin-like growth factors and phosphatidylinositol 3-kinase. The Journal of biological chemistry.

[25] Schaafsma D (2007). Insulin increases the expression of contractile phenotypic markers in airway smooth muscle. American journal of physiology. Cell physiology.

[26] Gosens R (2003). Insulin induces a hypercontractile airway smooth muscle phenotype. Eur J Pharmacol.

[27] Dekkers BG, Schaafsma D, Tran T, Zaagsma J, Meurs H (2009). Insulin-induced laminin expression promotes a hypercontractile airway smooth muscle phenotype. Am J Respir Cell Mol Biol.

[28] Wohlsen A (2003). The early allergic response in small airways of human precision-cut lung slices. Eur Respir J.

[29] Seehase S (2011). Bronchoconstriction in nonhuman primates: a species comparison. J Appl Physiol (1985).

[30] Akram KM (2019). Live imaging of alveologenesis in precision-cut lung slices reveals dynamic epithelial cell behaviour. Nat Commun.

[31] Sturton RG, Trifilieff A, Nicholson AG, Barnes PJ (2008). Pharmacological characterization of indacaterol, a novel once daily inhaled 2 adrenoceptor agonist, on small airways in human and rat precision-cut lung slices. The Journal of pharmacology and experimental therapeutics.

[32] Wyatt TA (2012). Co-exposure to cigarette smoke and alcohol decreases airway epithelial cell cilia beating in a protein kinase Cepsilon-dependent manner. The American journal of pathology.

[33] Behrsing HP, Furniss MJ, Davis M, Tomaszewski JE (2013). & Parchment, R. E. *In vitro* exposure of precision-cut lung slices to 2-(4-amino-3-methylphenyl)-5-fluorobenzothiazole lysylamide dihydrochloride (NSC 710305, Phortress) increases inflammatory cytokine content and tissue damage. Toxicological sciences: an official journal of the Society of Toxicology.

[34] Belmonte KE, Jacoby DB, Fryer AD (1997). Increased function of inhibitory neuronal M2 muscarinic receptors in diabetic rat lungs. Br J Pharmacol.

[35] Gosens R, Zaagsma J, Meurs H, Halayko AJ (2006). Muscarinic receptor signaling in the pathophysiology of asthma and COPD. Respiratory research.

[36] Schaafsma D (2007). Insulin induces airway smooth muscle contraction. Br J Pharmacol.

[37] Singh S (2016). Hyperinsulinemia adversely affects lung structure and function. Am J Physiol Lung Cell Mol Physiol.

[38] Halayko AJ (1999). Divergent differentiation paths in airway smooth muscle culture: induction of functionally contractile myocytes. Am J Physiol.

[39] Mitchell RW, Halayko AJ, Kahraman S, Solway J, Wylam ME (2000). Selective restoration of calcium coupling to muscarinic M(3) receptors in contractile cultured airway myocytes. Am J Physiol Lung Cell Mol Physiol.

[40] Santos Cavaiola T, Edelman S (2014). Inhaled insulin: a breath of fresh air? A review of inhaled insulin. Clinical therapeutics.

[41] Terzano C (2009). Effect of insulin on airway responsiveness in patients with type 2 diabetes mellitus: a cohort study. The Journal of asthma: official journal of the Association for the Care of Asthma.

[42] Ceglia L, Lau J, Pittas AG (2006). Meta-analysis: efficacy and safety of inhaled insulin therapy in adults with diabetes mellitus. Annals of internal medicine.

[43] Vick A (2007). A 6-month inhalation study to characterize the toxicity, pharmacokinetics, and pharmacodynamics of human insulin inhalation powder (HIIP) in beagle dogs. Journal of aerosol medicine: the official journal of the International Society for Aerosols in Medicine.

[44] Belmonte KE, Fryer AD, Costello RW (1998). Role of insulin in antigen-induced airway eosinophilia and neuronal M2 muscarinic receptor dysfunction. J Appl Physiol (1985).

[45] Chen C (2012). Integrin alpha9beta1 in airway smooth muscle suppresses exaggerated airway narrowing. The Journal of clinical investigation.

[46] Kellner J (2007). IL-13Ralpha2 reverses the effects of IL-13 and IL-4 on bronchial reactivity and acetylcholine-induced Ca+ signaling. International archives of allergy and immunology.

[47] Jiang H (2012). Targeting phosphoinositide 3-kinase gamma in airway smooth muscle cells to suppress interleukin-13-induced mouse airway hyperresponsiveness. The Journal of pharmacology and experimental therapeutics.

[48] Singh S, Prakash YS, Linneberg A, Agrawal A (2013). Insulin and the lung: connecting asthma and metabolic syndrome. Journal of allergy.

[49] Kuperman DA (2002). Direct effects of interleukin-13 on epithelial cells cause airway hyperreactivity and mucus overproduction in asthma. Nat Med.

[50] Paez-Cortez J (2013). A new approach for the study of lung smooth muscle phenotypes and its application in a murine model of allergic airway inflammation. PloS one.

[51] Charan J, Kantharia ND (2013). How to calculate sample size in animal studies?. Journal of pharmacology & pharmacotherapeutics.

